# Impact of maturity stage and prolongation of post-harvest processing and mucilage fermentation time on mycotoxin levels in coffee

**DOI:** 10.3389/fpls.2026.1734522

**Published:** 2026-02-02

**Authors:** Valentina Osorio, Esther Cecilia Montoya, Lina María Rayo-Mendez, Gabriel Keith Harris

**Affiliations:** 1 Quality Department, National Coffee Research Center, FNC, Manizales, Colombia; 2 Department of Food, Bioprocessing, and Nutrition Sciences, North Carolina State University, Raleigh, NC, United States

**Keywords:** mycotoxins, ochratoxin A, aflatoxin, fermentation, reserve, maturity stage

## Abstract

Different wet processing conditions generate changes in the quality characteristics of the coffee beans. Currently, the primary sources of these changes are the exocarp and mesocarp, and processing times are increased before pulping and during the fermentation of mucilage for this purpose. This research evaluated the mycotoxin contents of fruit at three different stages of maturity (MS1, MS2, and MS3), subjected to times and two temperatures of 15 and 20°C, under fruit reserve and prolonged fermentation conditions. No significant differences were detected at the maturity stage, with ochratoxin values of 2.89, 2.98, and 3.03 μg/kg for MS1, MS2, and MS3, respectively, and the average aflatoxin concentrations were 0.13 μg/kg, 0.14 μg/kg and 0.15 μg/kg, respectively. For the fruit reserve treatments associated with stages MS1, MS2 and MS3, the ochratoxin contents were 3.05, 3.19 and 3.27 μg/kg, respectively. The maximum observed for the reserve treatments was 3.31 μg/kg in MS3, with a temperature of 20°C and a reserve time of 48 hours. The maximum average value observed for the treatments with prolonged fermentation was 3.51 μg/kg in MS2, with a temperature of 20°C and a prolonged fermentation time of 20 hours. These values indicate that regardless of processing delays, the initial quality of the fruit is critical to ensure the safety of the bean. The elimination of dry and defective fruit through different classifications followed by continuous drying prevents the development of the conditions necessary for the generation of mycotoxins.

## 1 Introduction

Mycotoxins are toxic compounds originating from the secondary metabolism of specific fungal growth products, such as *Aspergillus* and *Penicillium* spp., that have different toxicological effects on human and animal health when ingested (Garcia-Moraleja et al., 2015). Several types of mycotoxins are commonly found in food, and the most prevalent mycotoxins are ochratoxin A (OTA) and aflatoxin B1 (AFB1), which are known for their nephrotoxic and hepatotoxic activities, respectively; therefore, they can cause a range of adverse effects, including organ damage (Attiya et al., 2021). Although both mycotoxins have health implications, OTA has been the primary focus of extensive studies because of its prevalence of metabolites and survival stability after high-temperature or low-pH conditions (Castellanos-Onorio et al., 2011; Oliveira et al., 2013; Paterson et al., 2014). OTA has been detected in several food products, such as cereals, oilseeds, coffee beans, meat, wine, cocoa, and spices (Vatinno et al., 2008).

Coffee fruits are susceptible to contamination by mycotoxigenic fungi either during harvest or postharvest processes (Garcia-Moraleja et al., 2015) or during transportation due to deficient sanitary conditions (Twaruzek et al., 2020). On the other hand, evidence has shown that these toxigenic fungi are present in both green and roasted coffee beans, both in producer and nonproducer areas of the world (Attiya et al., 2021; Galarce-Bustos et al., 2014; Twaruzek et al., 2020); thus, the ability of an OTA-producing strain to contaminate coffee beans depends on several factors, such as the environmental conditions and the type of processing or storage conditions (Benites et al., 2017).

Currently, the European Commission (EC) regulates OTA levels through law 1370/2022. The maximum allowable OTA content is 3 μg/kg in roasted coffee beans and 5 μg/kg in soluble coffee (Humaid et al., 2019). These levels mark a reduction from the 2006 resolution, which stipulated limits of 5 and 10 μg/kg, respectively. In terms of geographical origins, research on ochratoxin A has revealed that samples from American areas present significantly lower levels than those from African areas, a distinction attributed to differences in climatic and processing conditions (Benites et al., 2017).

Each fungal species has specific conditions for mycotoxin production. Not all visually altered beans are contaminated with this toxin, and conversely, not all visually healthy beans are free of mycotoxins (Heilmann et al., 1999). The total amount of available water and temperature are the most critical factors for the growth of OTA-producing fungi. In coffee, *Aspergillus ochraceus*, *A. carbonarius* and *A. niger* are responsible for OTA production (Gopinandhan et al., 2008; Taniwaki et al., 2014). *A. ochraceus* grows optimally at 30°C and produces OTA at 25–30°C, and *A. carbonarius* produces OTA at a higher rate at 30°C than at 20°C and grows optimally at 25–30°C (Paterson et al., 2014). Silva et al. (2008) reported that the minimum water activity (wa) required to produce ochratoxin is 0.85, which corresponds to a moisture content of approximately 20%. OTA-producing fungi are unlikely to grow at water activity (wa) values above 0.95 because hydrophilic fungi and yeasts will exceed them. These compounds can be present at an wa of 0.80 but do not produce toxins; they cannot grow below 0.78-0.76 (Paterson et al., 2014). To avoid OTA contamination, 12.5% moisture is the maximum water content in coffee. The presence of aflatoxin in green and roasted coffee beans is limited, with the primary producers being *A. flavus* and *Aspergillus parasiticus*. The optimal temperatures for aflatoxin production are 33°C and 35°C. Aflatoxin was detected during the later stages of fermentation, particularly during storage, with longer storage periods being more susceptible to contamination. Coffee from Guatemala was found to contain aflatoxin, and AFB1 contamination was detected in 1% of green coffee beans, ranging from 3 to 12 μg/kg (Paterson et al., 2014).

During harvesting and processing, different fungi and bacteria can contaminate coffee beans, but this type of contamination cannot represent a risk due to the application of high temperatures during roasting. However, mycotoxins have different levels of temperature resistance to OTA, and their contents can change during the coffee production process, resulting in variable reductions (from 22 to 90%) (Galarce-Bustos et al., 2014). Therefore, roasting procedures do not fully degrade OTA in green coffee beans, and most OTA is transmitted to beverages (Tsubouchi et al., 1987). Garcia-Moraleja et al. (2015) reported that coffee intake does not pose a potential risk to consumers concerning individual mycotoxin contamination, but further studies on mycotoxin concurrence in coffee are still needed.

The quantification of mycotoxins in the coffee matrix requires analytical methodologies that combine high sensitivity with detection limits below international regulatory thresholds. Currently, liquid chromatography coupled to mass spectrometry (LC-MS/MS) is established as the reference standard, due to its exceptional specificity and capacity for multiresidue analysis at trace levels (Vargas et al., 2022). Likewise, highly sensitive alternative technologies have emerged, such as electrochemical methods and biosensors, which stand out for their potential for portability and rapid response. However, for routine monitoring in the supply chain and the processing of a considerable volume of samples, immunochemical techniques such as the direct competitive enzyme-linked immunosorbent assay (CD-ELISA) offer a strategic competitive advantage. This method not only provides reliable correlation with chromatographic techniques, but also ensures high operational efficiency, facilitating timely decisions in postharvest quality control (Wacoo et al., 2014; Gashua et al., 2020).

The current trends in coffee consumption and the search for flavor innovations have prompted coffee producers to implement alterations throughout various phases of postharvest coffee processing. Currently, prevalent practices often prolong the processing duration of coffee cherries. This extended contact time between the coffee seeds and their surrounding skin and mucilage induces alterations that directly impact the sensory attributes of the resulting beverage. However, combining these methods with safety measures is crucial given that the conditions inherent to the fruit provide an environment conducive to the formation of mycotoxins. In the differentiation by processing, in the sensory quality of the samples from the fruit reserve, the cuppers identified 421 sensory flavor descriptors. Increasing the storage time from 24 to 48 hours showed a decrease in the frequency of descriptors from the caramel-sweet and chocolate groups, with a reduction in values from 53.57% to 46.42% and from 58.82% to 41.17%, respectively. The opposite behavior was observed for the descriptors of the fruit and red fruit groups, which increased their values from 33.33% to 66.66% and from 34.04% to 65.95%, respectively (Osorio et al., 2024). On the other part, in the treatments with prolonged fermentation, when increasing the temperature in the fruits of maturity stage MS3, which was the most advanced stage evaluated, a negative effect was evidenced, since it generated a decrease in the scores of five sensory attributes of the eleven evaluated (Osorio et al., 2022). The process temperature had an effect on the attributes of fragrance/aroma, residual flavor, acidity, balance and taster score, reducing their values from 7.62 to 7.40, 7.31 to 6.60, 7.41 to 6.65, 7.36 to 6.62 and 7.36 to 6.63, respectively. These decreases are associated with the values obtained in the MS3T20t20 treatment, which are in the lowest segment of the evaluation scale due to sensory defects.

There is a need to understand the relationship between mycotoxin growth and environmental conditions as well as processing conditions; therefore, this study evaluated mycotoxin levels by establishing conditions favorable for the growth of microorganisms associated with their production, such as nontraditional stages of wet processing, delays in the pulping of the fruit, and the prolongation of mucilage fermentation of coffee fruits at different maturation stages.

## 2 Materials and methods

### 2.1 Sample preparation

**Figure 1:**
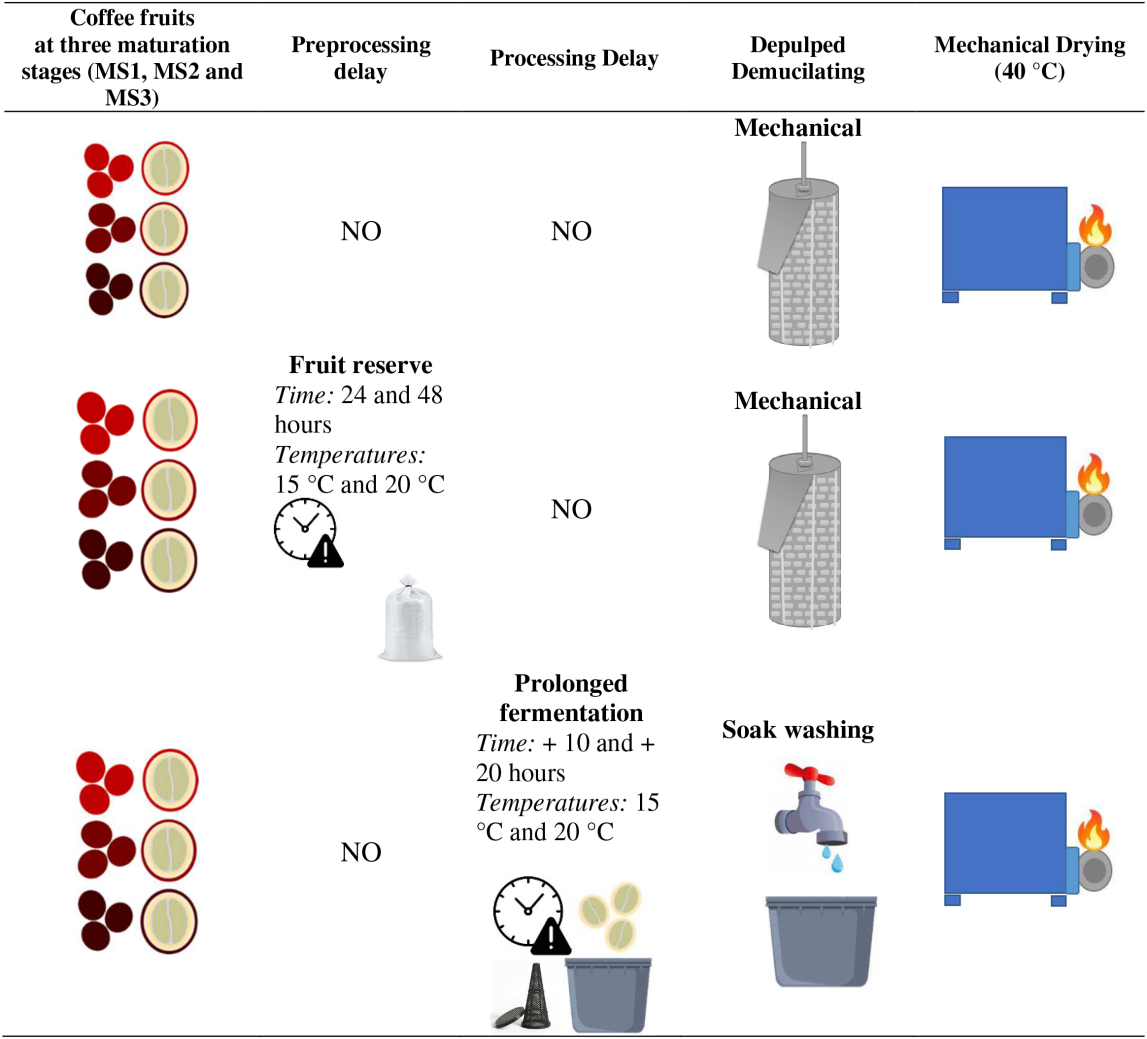
Flowchart illustrating the coffee processing workflow and experimental phases. Three coffee fruit maturity stages (MS1, MS2, and MS3) were evaluated at each processing condition. The first phase involved immediate processing without delay, including mechanical depulping followed by mechanical drying at 40 °C. The second phase incorporated a fruit reserve period of 24–48 h at 15–20 °C prior to depulping and drying under the same mechanical conditions. The third phase included a prolonged fermentation step lasting 10–20 h at 15–20 °C, followed by soak washing and mechanical drying. Importantly, all three maturity stages were subjected to each processing phase.

Coffee fruits (*Coffea arabica* L, Castillo^®^ variety, 800 kg) were manually harvested from coffee plantations in Chinchiná, Caldas, Colombia. The low-quality cherries with a density exceeding the water level were subsequently removed based on hydraulic classification. The coffee fruits were then categorized into three degrees of maturity, delineated as red–orange (MS1), crimson (MS2), and red wine (MS3), as illustrated in **Figure 1**.

### 2.2 Experimental design

To evaluate the level of mycotoxins and the impact of processing time delays (24 and 48 h) and temperatures (15 and 20°C) before pulping and during fermentation of coffee cherries at three different maturation stages (MS1, MS2, and MS3), a completely randomized experimental design with a 2x2 factorial configuration was applied. The experimental design is illustrated in **Figure 1**. Similarly, the same randomized design was used for prolonged fermentation, with additional fermentation times of 10 and 20 hours at two temperatures (15°C and 20°C).

### 2.3 Coffee fruits without processing delays

Eight working units of 200 kg of coffee cherries at three maturation stages were hydraulically sorted to separate low-quality fruits. The exocarp was removed using a 2500 horizontal pulper with a circular sieve (JM Estrada, Colombia) within less than 6 hours of harvest. The mucilage was then mechanically removed, followed by mechanical drying at 40°C using an air-forced dryer until the moisture content of the beans reached between 10 and 11.5% (wet basis).

### 2.4 Coffee fruits with delays before processing

In this research, delays in fruit processing establish the category of reserved coffees. Eight different working units were designed per maturity stage (80 kg) and divided into four subunits of 20 kg. In total, 12 treatments were used, with each consisting of 20 kg of coffee fruit stored in polypropylene bags with the following codes: reserve (R); maturity stage (MS); temperature (T) with values of 15°C and 20°C, and storage time (t) with values of 24 h and 48 h. The following 12 treatments were employed: RMS1T15t24, RMS1T15t48,RMS1T20t24, RMS1T20t48, RMS2T15t24, RMS2T15t48, RMS2T20t24, RMS2T20t48, RMS3T15t24, RMS3T15t48, RMS3T20t24, and RMS3T20t48 (**Table 1**). After fruit reserve processing (treatments), the coffee cherries from the 12 treatments were depulped, and the mucilage was removed and then dried under the same conditions as the fruits without processing delays (**Figure 1**).

**Table 1:** Treatment codes.

Processing Phases	MS1	MS2	MS3
		
*Coffee fruits without processing delays*	MS1 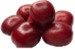	MS2 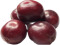	MS3 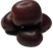
*Delays before processing (Temp 15 °C and 20 °C – Time 24 and 48 h)*	RMS1T15t24 RMS1T15t48 RMS1T20t24 RMS1T20t48	RMS2T15t24 RMS2T15t48 RMS2T20t24 RMS2T20t48	RMS3T15t24 RMS3T15t48 RMS3T20t24 RMS3T20t48
*Prolonged fermentation (Temp 15 °C and 20 °C – Time 10 and 20 h)*	FMS1T15t10 FMS1T15t20 FMS1T20t10 FMS1T20t20	FMS2T15t10 FMS2T15t20 FMS2T20t10 FMS2T20t20	FMS3T15t10 FMS3T15t20 FMS3T20t10 FMS3T20t20

### 2.5 Coffee fruits with prolonged mucilage fermentation.

Eight different working units of pulped coffee were designed per maturity stage (80 kg) and divided into four subunits of 20 kg. Fruits were pulled with a 2500 horizontal machine with a circular sieve (JM Estrada, Colombia). Twelve treatments were established and denoted using the following codes: fermentation (F); maturity stage (MS); temperature (T) with values of 15°C and 20°C; extension time (t) with values of 10 h and 20 h. This yielded the FMS1T15t10, FMS1T15t20, FMS1T20t10, FMS1T20t20, FMS2T15t10, FMS2T15t20, FMS2T20t10, FMS2T20t20, FMS3T15t10, FMS3T15t20, FMS3T20t10, and FMS3T20t20 treatments (**Table 1**), each with 20 kg of coffee fruits. Prolonged fermentation was established as follows: the fermentation process was monitored following the Fermaestro^TM^ methodology (Peñuela et al., 2010). Following the end time designated by this method, the coffee beans underwent an additional fermentation process for two additional times: 10 and 20 hours. After fermentation, the coffee beans were washed by soaking, sorting, and drying at 40°C to a moisture content ranging from 12 to 10% (**Figure 1**). In the eight units evaluated, the average initial time, as defined by the Fermaestro^TM^, was 16 hours, giving a total of 26 and 36 hours of process time in the prolonged fermentation.

### 2.6 Determination of ochratoxins and aflatoxins

Green coffee beans were cryogenically ground and stored at temperatures between 2 and -80°C until analysis. The total ochratoxin and aflatoxin contents were determined using a direct competitive enzyme-linked immunosorbent assay (CD-ELISA) with Veratox^®^ kits (Neogen, USA). The standard curve was prepared using ochratoxin controls of 0, 2, 5, 10, and 25 μg/L, and the aflatoxin controls used for 0, 5, 15 and 50 μg/L sample analyses were prepared according to the manufacturer’s methods. For ochratoxin extraction, 10.0 g of the sample was mixed with 40 mL of 50% (v/v) methanol/water mixture. In contrast, for aflatoxins, 5.0 g of ground sample was mixed with 25 mL of 70% methanol/water and vigorously agitated into small plastic jars with lids for two and three minutes, followed by filtration through a funnel and a Whatman^®^ qualitative filter paper, grade 1. The methanol used was of HPLC grade (Sigma-Aldrich), and the water used was of ultrapure quality (type I).

After extraction, 100 μL of conjugate solution was added to red-marked mixing wells, and 100 μL of control or extract samples were immediately added to each designated red-marked well. The samples were pipetted three times to mix the liquids, transferred to the microwells with antibodies, and incubated for 10 minutes at room temperature. The next step involved shaking the antibody wells, rinsing them five times with deionized water, removing the remaining water, turning the wells upside-down, and gently drying them with a paper towel. Then, 100 μL of substrate mixture was pipetted into the wells, which were subsequently incubated for ten minutes. Finally, 100 μL of red stop solution was added to the antibody well. The absorbance was measured at 450 nm using a multilabel reader (Perkin Elmer EnSpire 2300, USA) within 20 minutes after the addition of red stop solution. The concentration detected in the liquid extract was multiplied by the total volume of the extraction solvent and divided by the initial mass of the coffee sample. Additionally, a specific dilution factor was integrated to adjust the concentration to the sensitivity ranges of the calibration curve. This calculation allows transposing the analyte detected in the liquid phase to a mass per unit mass basis (μg/kg) of coffee, ensuring analytical traceability from sample preparation to the final result. The performance of the CD-ELISA method was validated internally to ensure the reliability of the results. The sensitivity of the assay was determined by the Limit of Detection (LOD) and Limit of Quantification (LOQ), which were 0.25 μg/kg and 0.75 μg/kg respectively. The calibration curve, constructed with six reference standards (0, 2, 5, 10, 25 and 50 μg/L), showed robust linearity with a coefficient of determination R^2^ > 0.95. The precision of the method was confirmed with intra-assay coefficients of variation (CV) less than 10%. Each treatment (27) had a total of eight samples (total of 216) and each sample was processed and analyzed in triplicate.

### 2.7 Water activity and moisture content

The water activity of the green coffee bean samples was determined with Lab Master Neo equipment (Novasina, Germany). The method was chosen when the equilibrium of the water activity value was not limited by the measurement time, and the stability of the reading was achieved when the variation did not exceed ± 0.003 for two minutes. The temperature was fixed at 25°C. The moisture content was measured using the standard direct method according to ISO 6673 at 105°C by the International Organization for Standardization, 2003 (VWR Scientific Forced-Air Oven 1350FMS). The measurements were performed in triplicate.

### 2.8 Statistical analysis

SAS 9.4 statistical software was used and the average and coefficient of variation by maturity stage, temperature and time were obtained. For each stage of maturity, an analysis of variance was performed with the response variables, corresponding to a completely randomized experimental design model in a 3*2*2 factorial arrangement with a significance level of α = 0.05. When the analysis of variance showed the effect of the treatments (p value <0.05), Duncan's multiple comparison test (α = 0.05) was performed on the treatments to identify the effect of temperatures and/or the times of maturity stage, reservation of the fruit or prolonged fermentation.

## 3 Results and discussion

### 3.1 Ochratoxin and aflatoxin contents at different maturity stages

**Figure 2:**
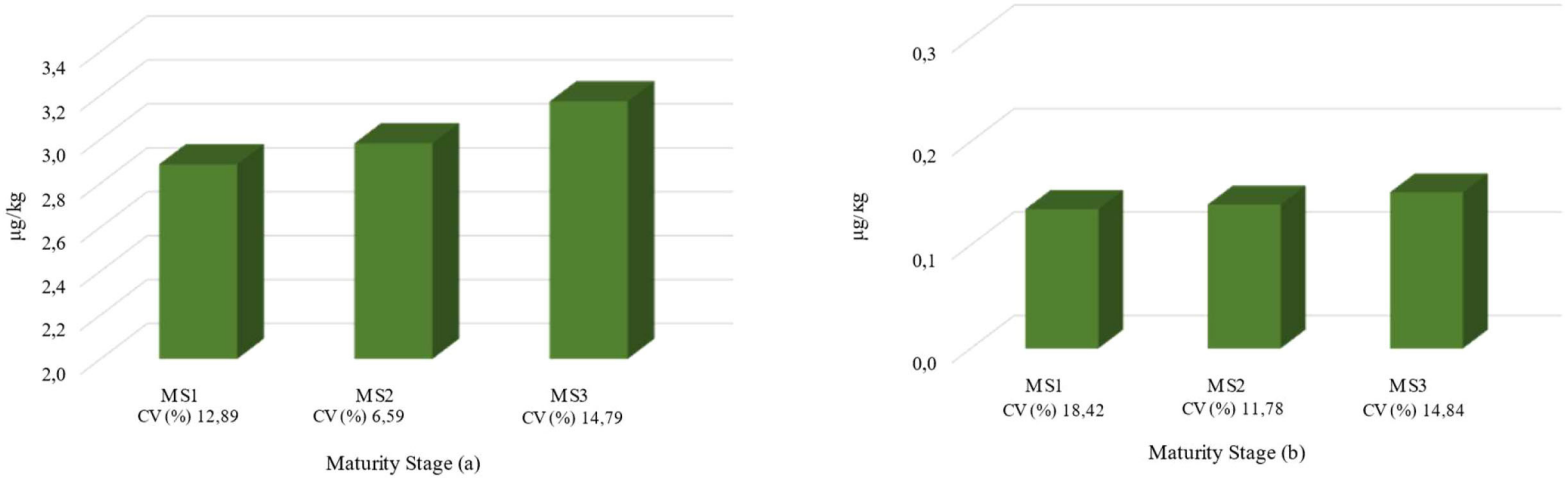
Bar charts illustrating mycotoxin concentrations across coffee fruit maturity stages (MS1, MS2, and MS3). Chart **(a)** presents ochratoxin levels expressed in μg/kg, with coefficients of variation (CV) of 12.89%, 6.59%, and 14.79% for MS1, MS2, and MS3, respectively. Chart **(b)** shows aflatoxin concentrations for the same maturity stages, also expressed in μg/kg, with corresponding CV values of 18.42%, 11.78%, and 14.84%.

Ochratoxin values of 2.89, 2.98 and 3.03 μg/kg for MS1, MS2 and MS3, respectively, were obtained. Of note, the values increased with increasing maturation stage, but no significant differences were detected (p>0.05). The minimum value of 2.54 μg/kg was found in the MS1 stage, and the maximum value of 4.04 μg/kg was found in the MS3 stage (**Figure 2**). The aflatoxin content was similar to the ochratoxin content, with the lowest values found at maturity stage MS1 and the highest at stage MS3. The average values for aflatoxin were 0.13 μg/kg, 0.14 μg/kg and 0.15 μg/kg for the MS1, MS2 and MS3 stages, respectively, with a minimum value of 0.10 in MS1 and a maximum value of 0.19 in MS3 (**Figure 2**). No significant differences were noted among the three stages evaluated.

According to Paterson et al., 2014, mycotoxin production is more significantly influenced by factors such as fruit quality, the presence of OTA-producing fungi, and environmental factors during drying than by specific drying procedures. In this study, it was developed under standardized processing conditions which guaranteed the optimum quality of the processed coffee fruits through various classifications applied at different post-harvest stages. This approach prevents the contact of healthy fruits with those of inferior quality, such as those damaged by insects and dried fruits. Batista et al. (2009) reported that fruits eliminated by hydraulic processes (floating coffee) have a high risk of OTA occurrence, consequently affecting the beans mixed with them. Additionally, in the three maturity stages evaluated in this study, the coffee drying process was subsequently performed, avoiding interruptions or prolonged periods that could create conditions, such as high moisture content in the beans, leading to fungal growth. However, Taniwaki et al. (2003) reported that an efficient drying process does not reduce OTA contamination. In contrast, OTA contamination depends on the ripeness of the fruit, with the most contaminated fruits being dry and defective. The MS3 maturity stage, considered an advanced stage, did not significantly differ from the MS1 stage. The contamination reported by other authors may be associated with the dry stages of the fruit, where there is a greater presence of beans with physical defects such as black and sour.

### 3.2 Reserve time

The average ochratoxin content for the reserve treatments associated with the MS1 stage was 3.05 μg/kg. At this maturity stage, the lowest average ochratoxin value of 2.93 μg/kg was observed in the treatment involving fruit reserves at a temperature of 15°C for 24 hours, whereas the highest average value of 3.19 μg/kg was found in the treatment with the longest reserve time and highest temperature, with values of 48 hours and 20°C (**Table 2**). The effects of the treatments associated with the MS2 maturity stage were similar to those associated with the MS1 maturity stage, with a mean value of 3.19. The lowest mean value of 3.08 μg/kg occurred in the treatment associated with fruit reserves at 15°C and 24 hours, whereas the highest mean value of 3.33 μg/kg was noted in the treatment with the longest reserve time and highest temperature, 48 hours and 20°C. The fruit reserve at the MS3 maturity stage presented a mean value of 3.27 μg/kg, representing the highest average value among the three maturity stages evaluated. The maximum mean value of 3.35 μg/kg, at the MS3 stage, was observed in the treatment associated with 20°C and 24 hours of reserve. The analysis of variance for ochratoxins in coffee fruits with reserve before processing revealed no significant effect on any of the evaluated factors: stage of maturity, time and temperature of the reserve.

**Table 2:** Ochratoxin and aflatoxin contents in coffee with fruit reserves.

Treatment	Ochratoxin (μg/kg)	CV (%)	Stand. error	Min	Max	Aflatoxin (μg/kg)	CV (%)	Stand. error	Min	Max
MS1-No reserve	2.89	12.89	0.13	2.54	3.57	0.13	18.42	0.01	0.10	0.18
RMS1T15t24	2.93	5.01	0.05	2.65	3.08	0.16	21.96	0.01	0.12	0.21
RMS1T15t48	2.98	7.19	0.08	2.63	3.20	0.16	20.51	0.01	0.12	0.22
RMS1T20t24	3.08	19.11	0.21	2.22	3.96	0.16	13.46	0.01	0.13	0.19
RMS1T20t48	3.19	14.55	0.16	2.58	3.82	0.16	12.18	0.01	0.14	0.20
MS2 -No reserve	2.98	6.59	0.07	2.70	3.26	0.14	11.78	0.01	0.12	0.17
RMS2T15t24	3.08	10.95	0.12	2.38	3.31	0.16	14.16	0.01	0.13	0.20
RMS2T15t48	3.16	11.22	0.13	2.82	3.74	0.16	16.56	0.01	0.12	0.20
RMS2T20t24	3.21	10.80	0.12	2.87	3.68	0.16	14.29	0.01	0.14	0.20
RMS2T20t48	3.33	7.26	0.09	2.97	3.65	0.16	20.42	0.01	0.12	0.21
MS3 -No reserve	3.17	14.79	0.17	2.63	4.04	0.15	14.84	0.01	0.12	0.19
RMS3T15t24	3.21	7.07	0.08	2.89	3.50	0.16	13.45	0.01	0.14	0.20
RMS3T15t48	3.23	14.75	0.17	2.74	3.93	0.16	23.54	0.01	0.12	0.23
RMS3T20t24	3.35	12.59	0.15	2.91	3.84	0.17	20.52	0.01	0.11	0.21
RMS3T20t48	3.31	9.60	0.11	2.83	3.84	0.17	23.01	0.01	0.12	0.23

Gopinandhan et al. (2008) reported that the type of coffee fruit processing influences OTA concentrations. Compared with wet-processed coffee, dry-processed coffee has a greater average content of mycotoxins, and the accumulation of mycotoxins in green beans from dried cherries is greater than that in dried parchment coffee. This is explained by the presence of the skin and mucilage of the fruit, which are optimal substrates for the growth of OTA-producing strains. Their removal during wet milling eliminates a suitable substrate for fungal growth. In this study, although the constituent parts of the fruit, such as the exocarp and mesocarp, had additional contact time with the bean, these parts were wet-processed, followed by continuous drying, which prevented the conditions necessary for mycotoxin generation from developing.

The average aflatoxin value in the treatments associated with the MS1, MS2 and MS3 maturity stages was 0.16 μg/kg. Specifically, in the MS1 maturity stage, the two reserve treatments at 15°C for 24 and 48 hours presented the lowest value of 0.12 μg/kg, whereas the highest value of 0.22 μg/kg was observed in the last treatment. For the MS2 maturity stage, minimum values of 0.12 μg/kg were observed in the 48-hour reserve treatments at both 15°C and 20°C, equal to the lowest levels observed in samples evaluated at MS2 without any reserve. In the MS3 state, the minimum aflatoxin value of 0.11 μg/kg was associated the 24-hour reserve treatment at 20°C, whereas the maximum value of 0.23 μg/kg was found in the 48-hour reserve treatment at 15°C and 20°C. Interestingly, similar to ochratoxins, aflatoxin levels are seemingly unaffected by variations in temperature, storage duration, or fruit maturity stage.

During reserve, the fruit temperature exhibited positive linear behavior. Fruits in maturity stages MS1 and MS3, which were stored at 15°C for 48 h, presented values of 30.8 and 38.02°C, which implied increases of 9.06°C and 13.58°C, respectively, with respect to the initial temperature. For these same maturity stages, in the treatments with reserves at 20°C, the additional 5° of the storage environment was reflected in the final temperature of the treatments. This is how the maturity stages MS1 and MS3 at hour 48 end, with average temperatures of 38.0°C and 40.48°C, respectively. Paterson et al. (2014) reported optimal temperatures of 33 and 35°C for the growth of *A. flavus* and aflatoxin production, which are higher than those of ochratoxigenic fungi. These same authors reported minimum and maximum temperature conditions ranging between 6 and 10°C and between 25 and 37°C, respectively, for the growth of *A. flavus* and additionally determined that higher humidity levels tend to favor this growth, defining a minimum water activity (wa) for aflatoxin production of 0.82. This corresponds to a moisture content of 18.4%. Although at this stage, a relatively high temperature of the fruit was evident, and the moisture content of the fruit was between 68% and 72% (wa close to 0.98), these conditions were not related to a relatively high concentration of aflatoxins in the coffee beans, possibly associated with the good quality of the fruit.

### 3.3 Prolonged fermentation

**Figure 3:**
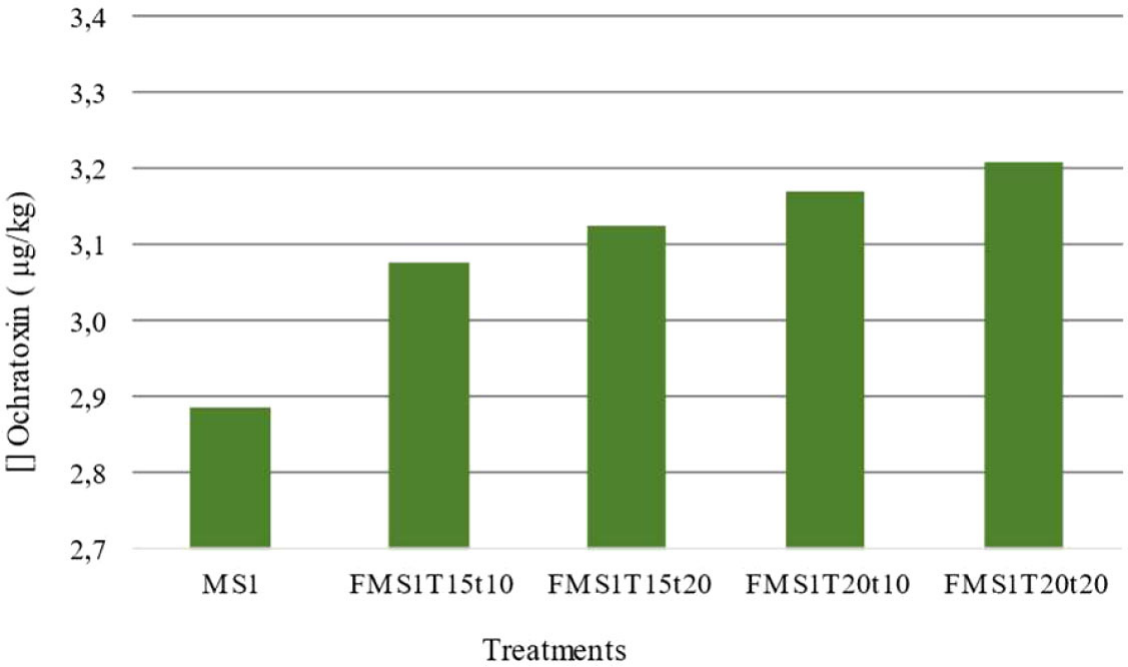
Bar chart illustrating ochratoxin concentrations (μg/kg) across the different processing treatments: MS1, FMS1T15t10, FMS1T15t20, FMS1T20t10, and FMS1T20t20. Ochratoxin levels ranged from 2.9 to 3.2 μg/kg, with treatment FMS1T20t20 exhibiting the highest concentration among the evaluated treatments.

Coffee fruits with previous hydraulic classification and separated into three different stages of maturity were removed and subjected to spontaneous fermentation. The 2x2 factorial design included two additional treatment durations as defined by the Fermaestro^TM^ methodology (10 and 20 hours) and two temperatures (15 and 20°C). The maturity stage MS1 presented a mean ochratoxin value of 3.14 μg/kg. The minimum mean value of 3.08 μg/kg was found in the treatment with a temperature of 15°C and an extension time of 10 hours, whereas the maximum mean value of 3.21 μg/kg was found in the treatment with a longer extension time (20 hours) and a higher process temperature (20°C) (**Figure 3**). For the treatments with different temperatures at the MS1 stage of maturity, a slight increase in ochratoxin concentration was observed from 10 hours to 20 hours of prolongation, but the analysis of variance revealed no effect on the ochratoxin concentration in the coffee bean from fruits at the MS1 stage of maturity due to the process temperature or any effect due to the prolongation of spontaneous fermentation.

Analysis of variance (ANOVA) revealed a significant effect of treatment on the ochratoxin content in fruits at the MS2 maturity stage (**Table 3**). Coffee derived from fruits at the MS2 maturity stage without prolonged fermentation presented an average ochratoxin content of 2.98 μg/kg and did not significantly differ (p > 0.05) from those in the treatments with a processing temperature of 15°C. These results, in fact, were different from those of the prolonged fermentation treatments with a processing temperature of 20°C, in which the average ochratoxin concentration was 15.92% greater. Throughout the spontaneous fermentation process, regardless of the processing temperature (15°C or 20°C), increases in mass temperature were observed. The most notable difference between treatments with different processing temperatures was 4.92°C at 24 h, which was reached at 36 h at 19.58°C and 23.02°C, respectively. The treatment with maturity stage MS3, process temperature of 20°C and 20 hours of prolongation presented a maximum mass temperature value of 27.30°C. The temperature increase is attributed to the exothermic process induced by microbial growth, and the temperatures achieved during the process decreased within the optimal ranges for the growth of most microorganisms. The optimal temperature for OTA production by A. *ochraceus* is in the range of 25–30°C, while A. *carbonarius* produces higher OTA concentrations at 30°C than at 20°C (Paterson et al., 2014). This variation may explain the higher ochratoxin concentrations observed in the treatments with a process temperature of 20°C.

**Table 3:** Ochratoxin contents in mature-stage MS2 coffee and prolonged fermentation.

Treatments	Ochratoxin (μg/kg) average		CV (%)	Standard error	Min	Max
MS2- No prolonged	2.98	B	6.59	0.07	2.70	3.26
FMS2T15t10	3.02	B	11.44	0.12	2.64	3.47
FMS2T15t20	3.01	B	11.03	0.12	2.66	3.68
FMS2T20t10	3.48	A	5.53	0.07	3.15	3.76
FMS2T20t20	3.51	A	11.16	0.14	2.94	3.94

Noncommon letters indicate differences in averages according to Duncan's test at 5% significance level.

The susceptibility of the MS2 maturity stage to temperature increase can be explained by the biochemical dynamics of mucilage during fruit processing. The fresh weight of the MS1 maturity stage is 10.14% higher than that of the MS3 stage, this loss of fresh weight between maturity stages 1 and 3 can be associated with the dehydration processes that the fruit undergoes in the last phase of ripening (Osorio et al., 2023). The mucilage contents in the maturity stages MS1 and MS2 are the same but are different from that of MS3; the maximum is found for MS1 with a value of 15.44, and the value decreases as the state of maturity increases until reaching 10.07. The MS2 maturity stage with 14,10% of mucilage, presents the most optimal balance between high water content and maximum accumulation of total soluble solids (TSS) in the mucilage. The MS1 stage has a higher moisture content and, on the contrast, in the MS3 stage, in spite of presenting concentrated TSS, the drastic reduction of free water content acts as a limiting factor (Osorio et al., 2023), restricting the proliferation of toxin-producing fungi.

**Figure 4:**
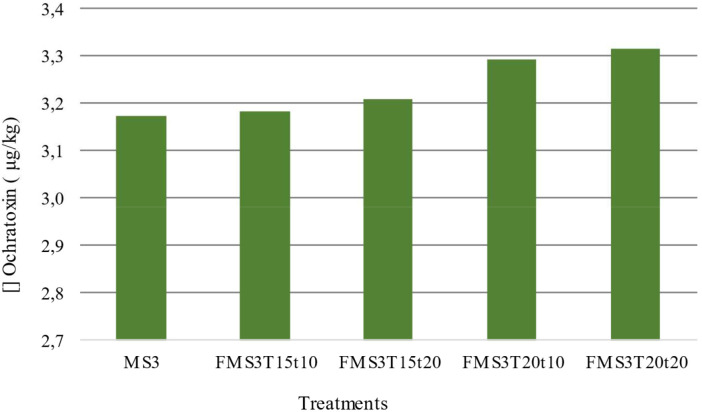
Bar chart illustrating ochratoxin concentrations (μg/kg) across five processing treatments: MS3, FMS3T15t10, FMS3T15t20, FMS3T20t10, and FMS3T20t20. Ochratoxin levels ranged from 3.1 to 3.3 μg/kg across all evaluated treatments.

Treatments associated with fruit at the MS3 maturity stage were not affected by the ochratoxin content. This concentration presented an average value of 3.25 μg/kg, and this value was equal to the average value of the treatments associated with the MS2 maturity stage. The difference in ochratoxin concentration between the treatments with 10 hours of fermentation and the different process temperatures (15°C and 20°C) was 0.11 μg/kg. This difference in process temperature for the treatments with a 20-hour duration was 0.10 μg/kg (**Figure 4**).

Regarding aflatoxin contents in samples from fruit at maturity stages MS1, MS2 and MS3, the analysis of variance revealed no effect of prolonged fermentation treatments (2x2 factorial design). The treatments associated with maturity stage MS1 presented a mean aflatoxin value of 0.17 μg/kg, and the minimum and maximum values observed were found in the treatments with a process temperature of 15°C with prolongation times of 10 and 20 hours. The first value was 0.14, and the last value was 0.23 μg/kg (**Table 4**). The treatments at the MS2 maturity stage presented the lowest average value among the maturity stages evaluated, at 0.16 μg/kg. For this maturity stage, the lowest values of 0.10 μg/kg were found in the 20°C treatments at the two extension times evaluated, whereas the maximum value of 0.22 μg/kg was observed in the two treatments with an extension time of 10 hours. For maturity stage MS3, the average of the treatments is the maximum among the evaluated maturity stages, with a value of 0.18 μg/kg. At MS3, the minimum and maximum values were found in the treatments with a temperature of 20°C. The minimum value of 0.14 μg/kg was noted at 20 hours, whereas the maximum value of 0.24 μg/kg was observed at these two prolonged times at the same process temperature.

**Table 4:** Aflatoxin content in coffee at different stages of maturity and prolonged fermentation.

Treatments	Aflatoxin (μg/kg) average	CV(%)	Standard error	Min	Max
MS1-No reserve	0.13	18.42	0.01	0.10	0.18
FMS1T15t10	0.17	19.45	0.01	0.14	0.23
FMS1T15t20	0.17	17.52	0.01	0.14	0.23
FMS1T20t10	0.17	12.90	0.01	0.15	0.22
FMS1T20t20	0.18	15.87	0.01	0.14	0.22
MS2-No reserve	0.14	11.78	0.01	0.12	0.17
FMS2T15t10	0.18	17.09	0.01	0.14	0.22
FMS2T15t20	0.16	16.92	0.01	0.11	0.20
FMS2T20t10	0.16	23.12	0.01	0.10	0.22
FMS2T20t20	0.16	22.55	0.01	0.10	0.21
MS3-No reserve	0.15	14.84	0.01	0.12	0.19
FMS3T15t10	0.18	7.60	0.01	0.16	0.20
FMS3T15t20	0.18	8.19	0.01	0.16	0.20
FMS3T20t10	0.18	17.16	0.01	0.15	0.24
FMS3T20t20	0.18	17.28	0.01	0.14	0.24

### 3.4 Moisture content and water activity

The average water activity (wa) for all treatments was 0.621, with a minimum average of 0.548 and a maximum average of 0.666; these values corresponded to average moisture values of 11.35%, 9.90% and 12.20%, respectively. No significant differences were found in moisture content or water activity among the treatments evaluated. For the maturity stages evaluated without any delay in processing, the average value was 0.622, with a corresponding moisture value of 11.30%. The maximum average water activity value of 0.648 was found in MS1, and the minimum average value of 0.642 was found in MS3. At the same maturity stage, a maximum moisture value of 12.20% and a minimum moisture value of 10.04% were reported for stage MS1. In the reserve stage, the treatments presented an average water activity value of 0.617. The minimum average value of 0.605 was observed in the TRMS1T15t24 treatment, whereas the maximum average value of 0.641 was found in the TRMS3T20t24 treatment, with average moisture values of 10.95% and 11.59%, respectively.

During the prolonged fermentation stage of pulped coffee from fruits at different stages of maturity, the average water activity value was 0.624. A minimum average value of 0.610 was obtained with the FMS2T15t10 treatment, whereas the maximum average value of 0.634 was noted with FMS1T20t20, with minimum and maximum moisture values of 10.39% and 12.04%, respectively. The water activity values and moisture contents for each treatment are shown in **Table 5**.

**Table 5:** Moisture content on a wet basis and water activity (wa).

Treatments	Moisture (%)	Desvest moisture(%)	Wa	Desvest Wa
MS1	11.25	0.53	0.620	0.018
MS2	11.26	0.39	0.631	0.021
MS3	11.41	0.72	0.616	0.024
FPMS1T15t10	11.62	0.32	0.631	0.009
FPMS1T15t20	11.54	0.46	0.633	0.035
FPMS1T20t10	11.16	0.49	0.614	0.031
FPMS1T20t20	11.68	0.33	0.634	0.013
FPMS2T15t10	11.28	0.44	0.610	0.026
FPMS2T15t20	11.22	0.56	0.626	0.016
FPMS2T20t10	11.52	0.27	0.621	0.019
FPMS2T20t20	11.47	0.51	0.631	0.022
FPMS3T15t10	11.43	0.52	0.617	0.019
FPMS3T15t20	11.11	0.59	0.623	0.015
FPMS3T20t10	11.58	0.40	0.625	0.020
FPMS3T20t20	11.19	0.62	0.628	0.028
TRMS1T15t24	10.95	0.52	0.605	0.027
TRMS1T15t48	11.24	0.62	0.615	0.033
TRMS1T20t24	11.35	0.44	0.614	0.021
TRMS1T20t48	11.10	0.42	0.606	0.020
TRMS2T15t24	11.28	0.43	0.616	0.024
TRMS2T15t48	11.38	0.64	0.616	0.026
TRMS2T20t24	11.43	0.42	0.623	0.031
TRMS2T20t48	11.44	0.43	0.619	0.025
TRMS3T15t24	11.26	0.39	0.617	0.021
TRMS3T15t48	11.17	0.52	0.608	0.021
TRMS3T20t24	11.59	0.32	0.641	0.013
TRMS3T20t48	11.62	0.42	0.618	0.031

Water content is an important factor in the shelf-life of foods. It contributes to their structure, and its interaction in the environment affects their relative stability during storage. It has been determined that lower wa values correspond to lower water availability to promote spoilage chemical reactions (Labuza, 2014). After processing delays were applied, all the samples analyzed were in the optimum moisture range (10%-12%) (**Table 5**). The variables of the mechanical drying process, such as temperature and flow rate, were controlled to ensure the optimal range of water content in the bean and thus avoid the configuration of possible physical defects, such as floppy for wet coffee or crystallization for overdried coffee. The wa and temperature conditions are important limiting factors for toxigenic fungal strain growth and ochratoxin production (Mannaa et al., 2018). The average values of the evaluated wa coffee samples were lower than those required for ochratoxin production. The values for OTA production range from 0.90 to 0.99, depending on the strain and growth conditions (Esteban et al., 2006). The wa interval for OTA (Ochratoxin A) production is smaller than that required for the growth of the fungi responsible for its production.

**Figure 5:**
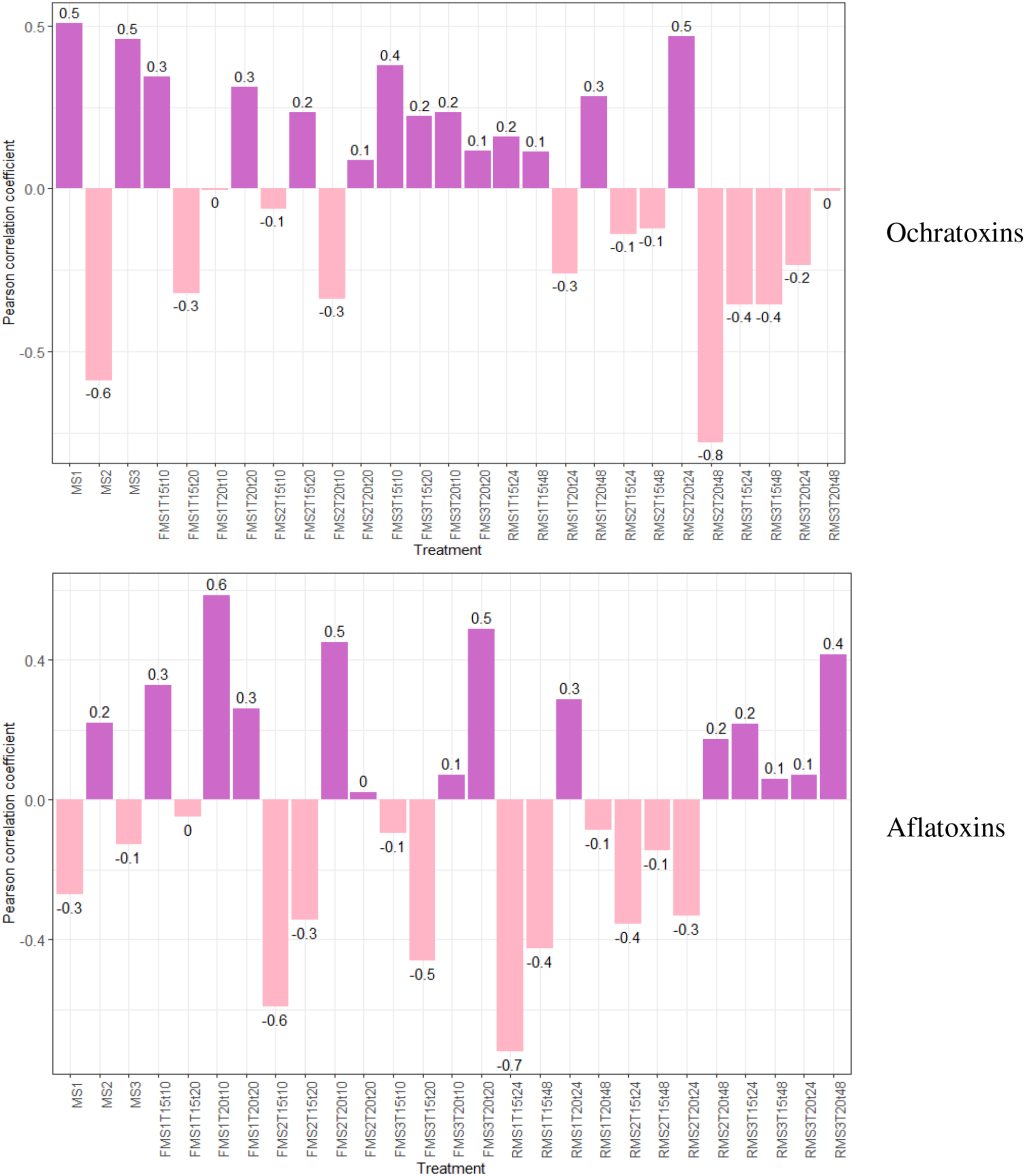
Bar charts comparing Pearson correlation coefficients for ochratoxins and aflatoxins across the different processing treatments. Both positive and negative correlations are presented, with numerical values indicated for each treatment.

To verify the relationships between mycotoxins and water activity (wa), the Pearson correlation coefficient based on treatment was determined (**Figure 5**). This coefficient is a measure of linear dependence between two quantitative random variables. If the value is 1, there is a perfect positive correlation. If the value is -1, there is a perfect negative correlation. Maturity stages MS1 and MS3 exhibited a positive correlation between wa and ochratoxin content, and this opposite relationship was noted for maturity stage MS2. This same correlation was evident in the treatments associated with fruit reserves before wet processing. With respect to the maturity stages and the correlation between wa and aflatoxin content, the behavior is different. Specifically, a positive correlation is noted for MS2, whereas a negative correlation is noted for MS1 and MS3. This might suggest that the conditions at this stage of maturity are more favorable for the growth of microorganisms associated with aflatoxin production. For the coffee beans, when the drying process is performed to ensure a moisture content in the range of 10% to 12%, a decrease in the risk conditions increases the mycotoxin content in the coffee.

Pardo et al. (2005) studied the influence of water activity and temperature on the germination and mycelial growth of three ochratoxigenic isolates of *Aspergillus ochraceus* and reported that the optimum conditions for germination and growth were 0.95–0.99 wa and 20–30°C, respectively. The germination and growth of *A. ochraceus* on green coffee beans can be prevented or inhibited when coffee beans are exposed to these temperature and humidity conditions during processing and storage.

## 4 Conclusion

Under the conditions evaluated, this research revealed that changes in the ochratoxin concentration in beans varied during the MS2 stage of maturity due to fluctuations in processing temperature during prolonged fermentation. However, this variation was not noticeable at the same temperature in whole fruit subjected to up to 48 hours of processing delay. This suggests that despite processing delays, the initial quality of the fruit is crucial for ensuring bean safety. The removal of dry and defective fruit using sorting methods and continuous drying prevents the conditions necessary for mycotoxin formation. When proper follow-up procedures are implemented during the drying process of coffee beans, ensuring a moisture content between 10% and 12%, the risk of conditions that promote mycotoxin formation is reduced. Like those of ochratoxins, aflatoxin levels are seemingly unaffected by variations in temperature, reserve duration, or fruit maturity stage.

## Data Availability

The original contributions presented in the study are included in the article/supplementary material. Further inquiries can be directed to the corresponding author.

## References

[B1] Attiya W. Hassan Z. U. Al-thani R. Jaoua S. (2021). Prevalence of toxigenic fungi and mycotoxins in Arabic coffee ( Coffea arabica ): Protective role of traditional coffee roasting, brewing and bacterial volatiles. PLOS ONE, 16 (10), e0259302. doi: 10.1371/journal.pone.0259302, PMID: 34714880 PMC8555823

[B2] Batista L. R. Chalfoun S. M. Silva C. F. Cirillo M. Varga E. A. Schwan R. F. (2009). Ochratoxin A in coffee beans (Coffea arabica L.) processed by dry and wet methods. Food Control 20, 784–790. doi: 10.1016/j.foodcont.2008.10.003

[B3] Benites A. J. Fernandes M. Rita A. Azevedo S. Silva S. Leitao A. L. (2017). Occurrence of ochratoxin A in roasted coffee samples commercialized in Portugal. Food Control 73, 1223–1228. doi: 10.1016/j.foodcont.2016.10.037

[B4] Castellanos-Onorio O. Gonzalez-rios O. Guyot B. Fontana T. A. Guiraud J. P. (2011). Effect of two different roasting techniques on the Ochratoxin A (OTA) reduction in coffee beans ( Coffea arabica ). Food Control 22, 1184–1188. doi: 10.1016/j.foodcont.2011.01.014

[B5] Esteban A. Abarca M. L. Bragulat M. R. Cabañes F. J. (2006). Effect of water activity on ochratoxin A production by Aspergillus Niger aggregate species. Int. J. Food Microbiol. 108(2), 188–195. doi: 10.1016/j.ijfoodmicro.2005.12.002, PMID: 16443301

[B6] Galarce-Bustos O. Alvarado M. Vega M. Aranda M. (2014). Occurrence of ochratoxin A in roasted and instant coffees in Chilean market. Food Control 46, 102–107. doi: 10.1016/j.foodcont.2014.05.014

[B7] Garcia-Moraleja A. Font G. Mañes J. Ferrer E. (2015). Analysis of mycotoxins in coffee and risk assessment in Spanish adolescents and adults. Food Chem. Toxicol. 86, 225–233. doi: 10.1016/j.fct.2015.10.014, PMID: 26514696

[B8] Gashua I. B. Williams S. I. Gashua M. M. (2020). Comparison of ELISA and HPLC for the determination of mycotoxins in stored commodities. J. Food Res. 9, 44–55. doi: 10.5539/jfr.v9n3p44

[B9] Gopinandhan T. N. Kannan G. S. Panneerselvam P. Velmourougane K. Raghuramulu Y. (2008). Survey on ochratoxin A in Indian green coffee destined for export. Food Additives Contaminants 1, 51–57. doi: 10.1080/19393210802236984, PMID: 24784537

[B10] Heilmann W. Rehfeldt A. G. Rotzoll F. (1999). Behaviour and reduction of ochratoxin A in green coffee beans in response to various processing methods. Eur. Food Res. Technol. 209, 297–300. doi: 10.1007/s002170050497

[B11] Humaid A. A. H. Alghalibi S. M. S. Al-Khalqi E. A. A. (2019). Aflatoxins and ochratoxin A content of stored Yemeni coffee beans and effect of roasting on mycotoxin contamination. Mol. Microbiol. 2, 11–21.

[B12] Labuza T. P. (2014). “ Interpretation of sorption data in relation to the state of constituent water,” in Water relations of foods ( Academic Press Inc), 155–172). doi: 10.1016/b978-0-12-223150-6.50014-6

[B13] Mannaa M. Kim K. D. Mannaa M. Kim K. D. (2018). Influence of Temperature and Water Activity on Deleterious Fungi and Mycotoxin Production during Grain Storage Mycobiology Influence of Temperature and Water Activity on Deleterious Fungi and Mycotoxin Production during Grain Storage. Mycobiology, 45(4), 240–254. doi: 10.5941/MYCO.2017.45.4.240, PMID: 29371792 PMC5780356

[B14] Oliveira G. da Silva D. M. Alvarenga Pereira R. G. F. Paiva L. C. Prado G. Batista L. R. (2013). Effect of different roasting levels and particle sizes on ochratoxin A concentration in coffee beans. Food Control 34, 651–656. doi: 10.1016/j.foodcont.2013.06.014

[B15] Osorio V. Álvarez C. I. Matallana L. G. Acuña J. R. Echeverri L. F. Imbachí L. C. (2022). Effect of prolonged fermentations of coffee mucilage with different stages of maturity on the quality and chemical composition of the bean. Fermentation 8, 519. doi: 10.3390/fermentation8100519

[B16] Osorio V. Matallana L. G. Fernandez-Alduenda M. R. Alvarez Barreto C. I. Gallego Agudelo C. P. Montoya Restrepo E. C. (2023). Chemical composition and sensory quality of coffee fruits at different stages of maturity. Agronomy 13, 1–15. doi: 10.3390/agronomy13020341

[B17] Osorio V. Pabón J. Medina R. (2024). Impact of fruit reserve conditions from harvest to processing on the chemical composition and quality of coffee. Front. Sustain. Food Syst. 8. doi: 10.3389/fsufs.2024.1425599

[B18] Pardo E. Ramos A. J. Sanchis V. Marı S. (2005). Modelling of effects of water activity and temperature on germination and growth of ochratoxigenic isolates of Aspergillus ochraceus on a green coffee-based medium. Int. J. Food Microbiol. 98, 1–9. doi: 10.1016/j.ijfoodmicro.2004.05.003, PMID: 15617796

[B19] Paterson R. R. M. Lima N. Taniwaki M. H. (2014). Coffee, mycotoxins and climate change. Food Res. Int. 61, 1–15. doi: 10.1016/j.foodres.2014.03.037

[B20] Peñuela A. E. Oliveros Tascón C. E. Sanz Uribe J. R. (2010). Remoción del mucílago de café a través de fermentación natural. Cenicafé 61, 159–173.

[B21] Silva C. F. Batista L. R. Schwan R. F. (2008). Incidence and distribution of filamentous fungi during fermentation, drying and storage of coffee (Coffea arabica L.) beans. Braz. J. Microbiol. 39, 521–526. doi: 10.1590/S1517-83822008000300022, PMID: 24031259 PMC3768428

[B22] Taniwaki M. H. Pitt J. I. Teixeira A. A. Iamanaka B. T. (2003). The source of ochratoxin A in Brazilian coffee and its formation in relation to processing methods. Int. J. Food Microbiol. 82, 173–179. doi: 10.1016/s0168-1605(02)00310-0, PMID: 12568757

[B23] Taniwaki M. H. Teixeira A. A. Teixeira A. R. R. Copetti M. V. Iamanaka B. T. (2014). Ochratoxigenic fungi and ochratoxin A in defective coffee beans. FRIN 61, 161–166. doi: 10.1016/j.foodres.2013.12.032

[B24] Tsubouchi H. Terada H. Yamamoto K. Hisada K. Sakabe Y. (1987). A survey of occurrence of mycotoxins and toxigenic fungi in imported green coffee beans, Mycotoxicol Y. Sakabe, Caffeine degradation and increased ochratoxin A production by toxigenic strains of Aspergillus ochraceus isolated from green coffee beans. Mycotoxins, 1984 (19), 16–21. doi: 10.2520/myco1975.1984.16

[B25] Twaruzek M. Kosicki R. Kwiatkowska-Gizynska J. Grajewski J. Altyn I. (2020). Toxicon Ochratoxin A and citrinin in green coffee and dietary supplements with green coffee extract. Toxicon 188, 172–177. doi: 10.1016/j.toxicon.2020.10.021, PMID: 33096150

[B26] Vargas E. A. Santos E. A. Whitaker T. B. (2022). Challenges and new perspectives in mycotoxin analysis of coffee: A review. Food Control 133, 108595. doi: 10.1016/j.foodcont.2021.108595

[B27] Vatinno R. Aresta A. Zambonin C. G. Palmisano F. (2008). Determination of Ochratoxin A in green coffee beans by solid-phase microextraction and liquid chromatography with fluorescence detection. Chromatogr. A 1187, 145–150. doi: 10.1016/j.chroma.2008.02.020, PMID: 18308329

[B28] Wacoo A. P. Wendiro D. Vuzi P. C. Hawumba J. F. (2014). Methods for detection of aflatoxins in agricultural food crops. J. Appl. Chem. 2014, 706291. doi: 10.1155/2014/706291

